# The Role of Acupoint Application of Herbal Medicine for Asthma: Meta-Analysis of Randomized Double-Blind Placebo-Controlled Trials

**DOI:** 10.1155/2022/5589433

**Published:** 2022-01-31

**Authors:** Zhenhu Wu, Yanchan Zheng, Yaoxin Chen, Bing He, Lei Wu, Lin Lin, Yinji Xu, Yuanbin Chen

**Affiliations:** ^1^State Key Laboratory of Dampness Syndrome of Chinese Medicine, The Second Affiliated Hospital of Guangzhou University of Chinese Medicine, Guangzhou 510120, China; ^2^Department of Respiratory Medicine, The Second Clinical College of Guangzhou University of Chinese Medicine and Guangdong Provincial Hospital of Chinese Medicine, Guangzhou 510120, China; ^3^Department of Respiratory Medicine, Shantou Hospital of Traditional Chinese Medicine, Shantou 515000, China; ^4^Clinical Laboratory, South China Normal University Hospital, Guangzhou 510630, China; ^5^Guangdong-Hong Kong-Macau Joint Lab on Chinese Medicine and Immune Disease Research, Guangzhou University of Chinese Medicine, Guangzhou, China

## Abstract

**Background:**

Acupoint application of herbal medicine (AAHM) has been widely used in China. At present, there is no systematic review of AAHM versus placebo in the treatment of asthma. This systematic review aims to assess the efficacy of AAHM for asthma.

**Methods:**

Searches were conducted in five English databases and four Chinese databases from their inceptions until December 2020. Randomized double-blind placebo-controlled trials were screened, and included studies evaluated routine pharmacotherapy (RP) plus AAHM versus RP plus placebo or AAHM versus placebo. The Cochrane risk of bias tool and Grading of Recommendations Assessment, Development and Evaluation (GRADE) were performed to evaluate the methodological quality and quality of evidence separately.

**Results:**

Sixteen studies involving 1,730 participants were included in this review. Compared with placebo plus RP, participants receiving long-term AAHM plus RP showed improvement in asthma quality of life questionnaire (AQLQ) with moderate-quality evidence (MD 6.53 points, 95% CI 2.70 to 10.36). Low-quality evidence indicated that AAHM plus RP was associated with improved FEV_1_ (%) compared with placebo plus RP, whether long- or short-term use (MD 11.80%, 95% CI 2.84 to 20.76; MD 10.57%, 95% CI 8.40 to 12.74; respectively). Moderate-quality evidence showed that participants receiving short-term AAHM were associated with a higher AQLQ score (MD 6.57 points, 95% CI 3.76 to 9.38) and a lower frequency of acute exacerbations (MD –1.84, 95% CI –2.32 to –1.36) compared with placebo. Low-quality evidence also indicated that AAHM was associated with improved FEV_1_ (L) compared with placebo, whether long- or short-term use (MD 0.35 litres, 95% CI 0.03 to 0.67; MD 0.66 litres, 95% CI 0.59 to 0.73; respectively).

**Conclusions:**

Moderate-quality evidence is promising that AAHM can improve the quality of life and reduce acute exacerbations in patients with asthma. AAHM also shows a positive role in improving lung function, but the evidence is so indefinite due to low quality.

## 1. Introduction

With the rapid advance of urbanization and industrialization leading to air pollution and lifestyle changes, the global incidence of asthma has increased. Currently, there are about 300 million people with asthma, and the number may reach 400 million by 2025 [1]. Asthma is not only a serious threat to human health but also a cause of enormous social and economic burden [2, 3]. Acupoint application of herbal medicine (AAHM) is a commonly used traditional Chinese medicine (TCM) technique for asthma [4]. AAHM is based on TCM meridian theory and involves the application of herbal paste on specific acupuncture points (also called point sticking therapy and acupoint plaster therapy). To make the paste, herbal medicines with antiasthmatic properties are ground into powder; reconciled into a paste; made into an ointment, pill, or pie; and then directly applied to acupoints. The paste is left on the acupoints for several hours, and then a new paste is applied every few days for weeks or months.

International conventional medicine guidelines recommend inhaled corticosteroids (ICS) for the long-term treatment of asthma [5]. However, long-term use can produce side effects such as increased fracture risk, osteoporosis, or oropharyngeal candidiasis [6]. Moreover, cessation of ICS after long-term use can cause symptom recurrence and increased risk of airway inflammation. Therefore, complementary and alternative therapies, including acupoint application, may be useful for the management of asthma.

Preclinical studies have demonstrated that AAHM can improve immunity, correct the TH1/TH2 proportional imbalance, and prevent the occurrence of airway inflammation and hypersensitivity, which are commonly seen in asthmatics [7, 8]. The goal of asthma treatment is to control asthma symptoms and maintain a normal level of activity while minimizing the risk of acute exacerbation and lung function damage [9]. A previous review of randomized controlled trials (RCTs) reported that AAHM plus routine pharmacotherapy (RP) compared to RP alone improved forced expiratory volume in 1 second (FEV_1_) and asthmatic symptoms in patients with asthma [10]. However, there is a lack of evidence for direct comparison with placebo and no assessment of the quality of the evidence. In addition, important outcomes such as asthma control and acute exacerbation frequency were not evaluated in the previous systematic review.

Recently, the number and quality of randomized double-blind placebo-controlled trials of AAHM for asthma have increased. Therefore, we systematically evaluated the evidence of multiple outcomes in order to determine whether adding AAHM was beneficial for patients with asthma.

## 2. Methods

### 2.1. Search Strategy

The following English and Chinese databases were searched from their inceptions until December 2020: PubMed, Allied and Complementary Medicine Database (AMED), Cumulative Index of Nursing and Allied Health Literature (CINAHL), Excerpta Medica Database (Embase), Cochrane Central Register of Controlled Trials (CENTRAL), Chongqing VIP (CQVIP), Chinese Biomedical Database (CBM), Wanfang Medicine Online (WFMO), and China National Knowledge Infrastructure (CNKI). The date and language of publication were not restricted. We also manually searched existing meta-analyses and scanned cited references in published studies to identify potentially relevant trials.

This strategy was intended to maximize the capture of citations for peer-reviewed publications relevant to three groups of search terms: condition (asthma and its synonyms); intervention (acupoint application and its synonyms), and study type (RCTs and its synonyms).

### 2.2. Inclusion Criteria

Studies were eligible if they satisfied the following criteria: (1) they were randomized double-blind placebo-controlled trials; (2) patients had been diagnosed with asthma according to clinical symptoms and pulmonary function tests (e.g., Global Initiative for Asthma [11] or the Chinese Medical Association's diagnosis and treatment guidelines [12]; (3) they compared RP plus AAHM versus RP plus placebo or AAHM versus placebo; (4) the medicine used in AAHM was Chinese herbal medicine. RP included corticosteroids, bronchodilators, theophylline, and leukotriene receptor antagonists; and (5) trials reported at least one of the following outcome measures: lung function including FEV_1_, forced vital capacity (FVC), asthma control test (ACT), asthma quality of life questionnaire (AQLQ), total immunoglobulin E (IgE), eosinophil (EOS), and/or frequency of acute exacerbations.

### 2.3. Exclusion Criteria

Studies were excluded if they included: (1) patients with chronic obstructive pulmonary disease, interstitial lung disease, or other respiratory diseases; (2) AAHM had been combined with either acupuncture, acupoint injection, oral Chinese herbal medicine, or other therapies; and (3) Trials had used antibiotics, oral, or intravenous steroids as RP treatment.

### 2.4. Data Extraction

Two independent reviewers (YXC and YCZ) screened the studies according to the eligibility criteria, and disagreements were resolved by a third researcher (YBC). Titles and abstracts were first screened, and unrelated articles were excluded. Then, full articles were identified based on predefined inclusion and exclusion criteria.

After screening the studies, data were evaluated and extracted by two researchers (ZHW and YCZ) using a standard form. The form included the following items: (1) general details (e.g., title, first author, publication date, diagnostic criteria, and duration of asthma); (2) participants (e.g., sample size, age, sex, and severity of asthma); (3) methodological evaluation; (4) experimental and control interventions; (5) outcome measures; (6) dropouts and treatment/follow-up duration; and (7) adverse events. Attempts were made to contact corresponding authors via e-mail, mail, or phone when data were missing or incomplete.

### 2.5. Risk of Bias Assessment

The methodological quality of the included studies was assessed independently by two reviewers (ZHW and YJX) using the Cochrane Collaboration's risk of bias assessment tool [13]. The risk of bias in seven domains was assessed: (1) random sequence generation; (2) allocation concealment; (3) blinding of participants and personnel; (4) blinding of outcome assessors; (5) incomplete outcome data; (6) selective outcome reporting; and (7) other bias such as baseline balance, funding source, and conflicts of interest. Each risk of bias domain was assessed as low risk, high risk, or unclear risk. Any discrepancies were resolved by discussion with a third reviewer (LW or LL).

### 2.6. Statistical Analysis

All statistical analyses were conducted using Review Manager (RevMan 5.3.3) from the Cochrane Collaboration. Continuous data were calculated as mean difference (MD), with 95% confidence intervals (CI). Visual inspection of forest plots, *p*-values, and *I*^2^ statistics were used to evaluate statistical heterogeneity. An *I*^2^ statistic over 50% (*I*^2^ ＞ 50%) was considered to be an indicator of high heterogeneity. If heterogeneity was low, a fixed-effects model was applied to estimate the treatment effect. If heterogeneity was high, a random-effects model was used. In addition, subgroup analysis and sensitivity analysis were used to explore potential heterogeneity. Funnel plot analysis was carried out to assess potential publication bias if there were ten or more studies in the pool.

### 2.7. Assessment of the Quality of Evidence

The Grading of Recommendations Assessment, Development, and Evaluation (GRADE) was used for developing and presenting summaries of evidence, and the quality of evidence in the meta-analysis was summarized and graded [14]. Although the quality of evidence is continuous, the GRADE method finally divided the quality of evidence group into four categories: high, moderate, low, and very low according to the level of evidence.

## 3. Results

### 3.1. Study Selection

**Figure 1:**
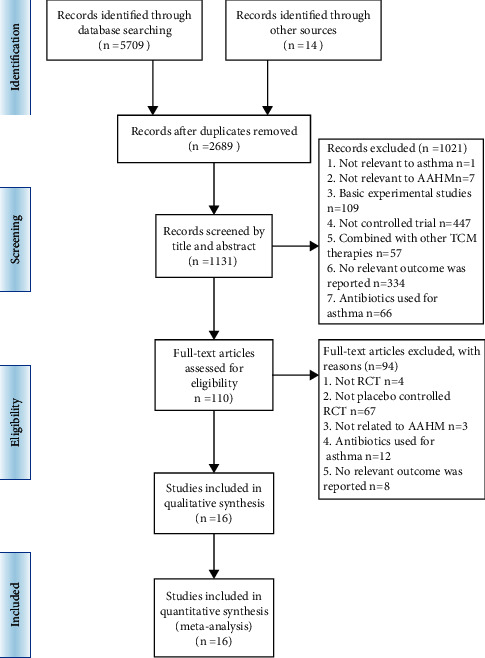
Flow diagram of the study selection process.

A total of 5,723 studies were initially retrieved from English and Chinese databases. After 3,034 duplicates were removed, the remaining 2,689 were screened. The full texts of 110 were retrieved after title and abstract screening. Full-text evaluation removed another 93 unrelated studies. A total of 16 eligible RCTs [15–30] (1,730 participants) were reviewed and included in the meta-analysis (Figure 1).

### 3.2. Study Characteristics

All studies [15–30] were conducted in China and retrieved from Chinese databases. In four studies [16–18, 20], the participants were children; 12 studies [15, 19, 21–30] included adults. Participants were diagnosed with asthma according to the Chinese Medical Association's diagnosis and treatment guidelines in all studies.

Ten studies [15, 18–26] applied the AAHM treatment during the hottest days of summer (San-Fu days also called dog days, between mid-July and mid-August); two studies [16, 17] were conducted during the hottest days of summer and the coldest days of winter (San-Jiu days, between early-January and mid-January); and four studies [27–30] did not mention the time of year of treatment. Herbal medicines included Xi Xin (Herbal Asari), Bai Jie Zi (Semen Brassicae), Gan Sui (Radix Kansui), Yan Hu Suo (Rhizoma Corydalis), and so on. Feishu (BL13), Dingchuan (EX-B1), Tiantu (CV22), Shenshu (BL23), and Dazhui (GV14) were commonly used as acupoints across included studies. RP included corticosteroids, bronchodilators, leukotriene receptor antagonist, and so on.

Participants enrolled in the studies were randomly divided into two groups: the treatment groups included AAHM alone or AAHM plus RP, and control groups included placebo AAHM alone or placebo plus RP. Six studies [15–17, 19, 25, 30] compared AAHM plus RP with placebo plus RP, and ten studies [18, 20–24, 26–29] compared AAHM alone with placebo alone. Treatment duration ranged from 4 weeks to 4 years. Studies with a treatment duration of 1 year or more were defined as long-term efficacy observation [15, 16, 19, 20, 22, 23, 25, 26], and those with a duration of less than 1 year were short-term efficacy observation [17, 18, 21, 24, 27–30]. Twelve studies [15–20, 23, 25, 27–30] reported lung function parameters (including at least one of FEV_1_ and FEV_1_/FVC); three studies [16, 19, 24] evaluated total IgE; four studies [15, 19, 21, 26] reported acute exacerbations; two studies [16, 20, 29] reported EOS; and three studies [25, 27, 28] assessed AQLQ; and two studies [15, 23] assessed ACT. Characteristics of all included studies are summarized in Tables 1 and 2.

### 3.3. Risk of Bias

Nine studies [15, 20–23, 25, 27–29] explicitly described methods for random sequence generation and were judged as having a low risk of selection bias. Two studies [23, 27] mentioned the methods of allocation concealment were judged to be at unclear risk. Sixteen studies [15–30] were judged at low risk of bias in the blinding of participants and personnel because acupoint application of placebo was reported. Incomplete outcome data were assessed at low risk if there was no missing data or dropouts were balanced between groups. Any studies without incomplete outcome data were judged to be at low risk. All studies were judged at low risk of bias for selective outcome reporting. Other biases including baseline imbalance, research funding, and conflicts of interest were at low risk in ten studies [16, 17, 21, 22, 24–26, 28–30]. The risk of bias is summarized in Figure S1.

### 3.4. Publication Bias

The tests for funnel plot asymmetry should not be used when there are fewer than ten studies in the meta-analysis. Therefore, we did not assess potential publication bias.

### 3.5. Results of AAHM plus RP versus Placebo plus RP

#### 3.5.1. Lung Function

Spirometry is used to demonstrate airway obstruction to confirm diagnosis as well as monitor asthma over time. Lung function FEV_1_ and FVC are the primary measures used to assess asthma. Increases of more than 0.23 litres or 10% indicate clinically important improvement [31, 32]. Improving lung function is one of the primary objectives of asthma management. To objectively measure asthma over time, regular monitoring of spirometry is commonly used in the clinical and research setting.

**Figure 2:**
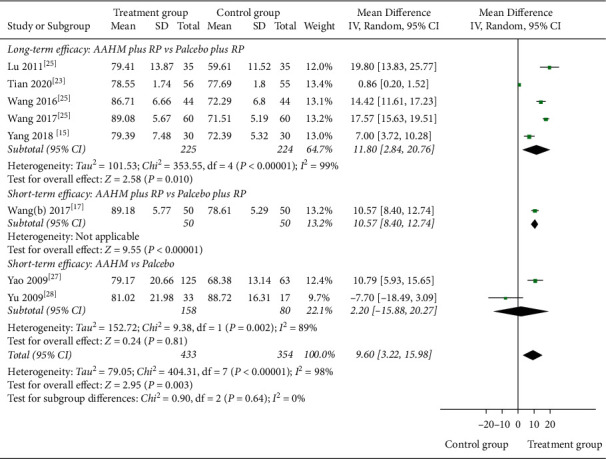
Forest plot of treatment group versus control group: FEV_1_ (%).

FEV_1_ (%) was assessed in six studies (549 participants) that were judged as low-quality evidence (Tables 3 and 4). The forest plot of long-term efficacy showed FEV_1_ (%) increased, although the heterogeneity was high (MD 11.80%, 95% CI 2.84 to 20.76, *I*^2^ = 99%), and the forest plot of short-term efficacy had a similar result (MD 10.57%, 95% CI 8.40 to 12.74; Figure 2). Low-quality evidence from one study evaluated the short-term efficacy of FEV_1_ (L; Table 4), and the result indicated that AAHM plus RP was better than placebo plus RP (MD 0.80 L, 95% CI 0.55 to 1.05; Figure S2).

FEV_1_/FVC was assessed in two studies (long-term efficacy) with very low-quality evidence (Table 3), and the forest plot showed no significant difference between the groups, and the heterogeneity was high (MD 9.77, 95% CI −4.75 to 24.28, *I*^2^ = 96%; Figure S3). However, another short-term efficacy observation with low-quality evidence showed that the treatment group was better than the control group in terms of FEV_1_/FVC (MD 7.77, 95% CI 6.07 to 9.47; Table 4 and Figure S3).

#### 3.5.2. Frequency of Acute Exacerbations

Exacerbations are characterized by acute worsening of wheezing, coughing, shortness of breath, chest tightness, and lung function reduction. Exacerbations put patients at risk of respiratory distress, hypoxemia, and airflow obstruction and are the most important marker of asthma management and treatment effectiveness [33]. Reduction of asthma exacerbation frequency is an important measure in asthma studies, and fewer exacerbations indicate better asthma control and reduced morbidity and mortality. Ideally, treatments should stabilize symptoms for long periods and prevent exacerbations.

**Figure 3:**
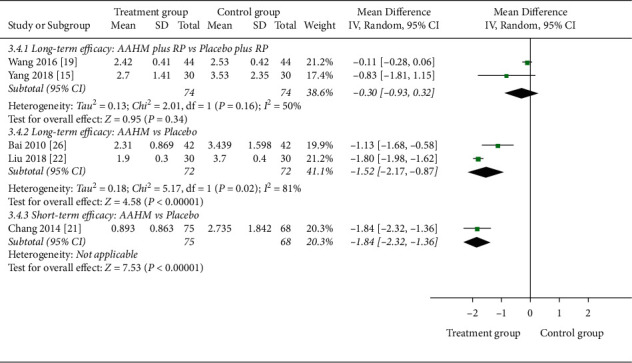
Forest plot of treatment group versus control group: frequency of acute exacerbations.

The frequency of acute exacerbations was evaluated in two studies (long-term efficacy) with very low-quality evidence (Table 3). The results showed AAHM plus RP was not better than placebo plus RP in reducing the frequency of acute exacerbations, and the heterogeneity was low (MD –0.30, 95% CI –0.93 to −0.32, *I*^2^ = 50%; Figure 3).

#### 3.5.3. Asthma Control Test

The ongoing assessment of patients with asthma is important, and symptom control is the goal of management. ACT was used to assess asthma control [34]. The ACT includes five items to assess shortness of breath, night-time waking, activity, rescue bronchodilator use, and rating of asthma control. Each item represents one question, including five answers, with a total of 25 points for all items [35]. A higher score indicates more controlled asthma.

**Figure 4:**
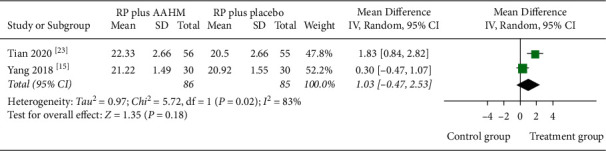
Forest plot of treatment group versus control group: ACT score.

Very low-quality evidence from two studies (171 participants) assessed the ACT (Table 3). The result of long-term efficacy showed no potential increase in ACT score (MD 1.03 points, 95% CI –0.47 to 2.53) when participants received AAHM plus RP, compared with those receiving placebo plus RP, and the heterogeneity was high *I*^2^ = 83% (Figure 4).

#### 3.5.4. Asthma Quality of Life Questionnaire

**Figure 5:**
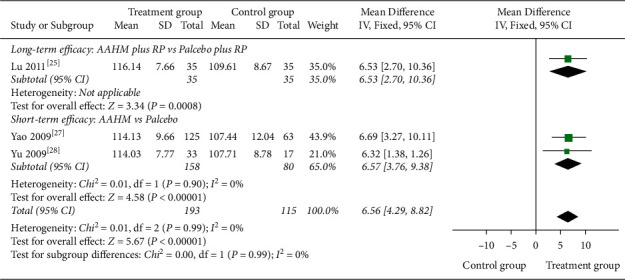
Forest plot of treatment group versus control group: AQLQ score.

Moderate-quality evidence from one RCT (70 participants) evaluated AQLQ (Table 3), and the result of long-term efficacy indicated that AAHM plus RP was superior to placebo plus RP in terms of improving AQLQ score (MD 6.53 points, 95% CI 2.70 to 10.36; Figure 5).

#### 3.5.5. Immunological Marker Total IgE

IgE is an immunological biomarker that plays an integral role in the pathogenesis of allergic diseases, including asthma [36]. The measurement of serum IgE is available in routine clinical practice and is used to define the biomarker status of patients with asthma.

Two studies (208 participants) with very low-quality evidence reported the total IgE when AAHM plus RP was compared to placebo plus RP (Table 3). The result of long-term efficacy showed treatment group was not superior to the control group at decreasing total IgE (Figure S4).

#### 3.5.6. Eosinophil

Low-quality evidence from 1 RCT with 120 participants assessed EOS (Table 3). The result of long-term efficacy showed that AAHM plus RP was better than placebo plus RP (MD –0.31, 95% CI −0.37 to −0.25; Figure S5).

### 3.6. Results of AAHM versus Placebo

#### 3.6.1. Lung Function

FEV_1_ (L) was assessed in two studies (173 participants) that were judged as low-quality evidence (Tables 5 and 6). Results showed that there was a significant difference between the groups, whether the long- or short-term efficacy (MD 0.35 litres, 95% CI 0.03 to 0.67; MD 0.66 litres, 95% CI 0.59 to 0.73, respectively; Figure S2). Very low-quality evidence from 2 studies including 238 participants (Table 6), assessed the short-term efficacy of FEV_1_ (%). The meta-analysis result indicated that there was no difference between AAHM and placebo (MD 2.20%, 95% CI –15.88 to 20.27, *I*^2^ = 89%; Figure 2).

Low-quality evidence from one study evaluated the long-term efficacy in improving the FEV_1_/FVC (Table 5), and the forest plot showed AAHM was better than placebo (MD 11.75%, 95% CI 4.04 to 19.46). Very low-quality evidence from two RCTs evaluated FEV_1_/FVC (Table 6), and the result of short-term efficacy indicated that AAHM was not superior to placebo (MD 0.68%, 95% CI –15.24 to 16.60; Figure S3).

#### 3.6.2. Frequency of Acute Exacerbations

The frequency of acute exacerbations was assessed in 3 studies (287 participants). The results of long-term efficacy showed AAHM was better than placebo in reducing the acute exacerbations, and the quality of evidence was very low (MD –1.52, 95% CI –2.17 to –0.87, *I*^2^ = 81%; Table 5 and Figure 3). Another short-term efficacy observation with moderate evidence showed that AAHM was also superior to placebo (MD –1.84, 95% CI –2.32 to –1.36; Table 6 and Figure 3).

#### 3.6.3. Asthma Quality of Life Questionnaire

Moderate-quality evidence from 2 studies (238 participants) assessed the AQLQ (Table 6). The result of short-term efficacy showed a potential increase in AQLQ score (MD 6.57 points, 95% CI 3.76 to 9.38) when participants received AAHM, compared with those receiving placebo alone, and the heterogeneity was low *I*^2^ = 0% (Figure 5).

#### 3.6.4. Immunological Marker Total IgE

Low-quality evidence from 1 RCT with 120 participants assessed total IgE (Table 6). The result of short-term efficacy showed the total IgE reduced in participants receiving AAHM compared to those receiving placebo (MD –104.40 IU/ml, 95% CI −113.14 to −95.66; Figure S4).

#### 3.6.5. Eosinophil

Low-quality evidence from two RCTs evaluated EOS (Tables 5 and 6). The result indicated that there was no difference between AAHM and placebo, whether the long- or short-term efficacy (Figure S5).

### 3.7. Adverse Events

The occurrence of adverse events was reported in three studies [15, 21, 23], and no serious adverse events were reported. These studies included 13 participants and reported adverse events associated with acupoint sticking. Adverse events associated with AAHM were judged to be of mild intensity and consisted of local skin redness, swelling, itching, or blisters. These symptoms were related to direct irritation caused by the herbal medicine and duration of acupoint application. Symptoms were relieved by reducing the acupoint application time, maintaining dryness, or coating the affected area with mupirocin antibiotic ointment.

## 4. Discussion

Asthma is an increasingly prevalent health problem worldwide; the prevalence of asthma among people aged 20 and over in China is 4.2%, with 45.7 million patients [1, 37]. AAHM is the topical application of Chinese herbal paste on acupuncture points. AAHM therapy for asthma has received widespread research attention over the past 30 years. Nowadays, AAHM as a complementary and alternative therapy has been widely used for asthma in East Asian countries, including China [38, 39]. AAHM not only has the effect of stimulating acupuncture points; the active ingredients of the herbs can be absorbed through the skin. It is relatively safe and easy to apply and has a dual therapeutic effect. It is also an external therapy that does not require oral or intravenous medication. Furthermore, it is popular with both adults and children.

The primary objectives of asthma management are to control symptoms, maintain lung function and activity levels, and prevent asthma exacerbations and mortality. Two systematic reviews reported positive therapeutic effects of AAHM for asthma. They suggest that it may contribute to improving pulmonary function, clinical symptoms and reducing interleukin and IgE levels [10, 40]. However, the authors found that not enough evidence was available to conclude whether or not AAHM should be recommended. They also suggested that further research is conducted to clarify the role of AAHM.

To our knowledge, this is the first meta-analysis of AAHM versus placebo in the treatment of asthma. It is different from previous systematic reviews because it evaluates randomized double-blind placebo-controlled trials and outcomes including lung function FEV_1_, AQLQ, ACT, acute exacerbations, total IgE, and EOS. Sixteen studies were included for this meta-analysis. Participants receiving RP plus AAHM or AAHM alone were compared with those receiving RP plus placebo or placebo alone. Moderate-quality evidence demonstrates that RP combined with AAHM or AAHM alone can improve the quality of life of asthma patients, and short-term use of AAHM was associated with a lower frequency of acute exacerbations. Moreover, very-low- to low-quality evidence also indicated that AAHM was associated with improved lung function and reduced total immunoglobulin E and EOS.

Chronic inflammation of the airways is the key feature of asthma. The inflammatory process is complex and involves many cells, such as eosinophils, T-lymphocytes, Mast cells, and macrophages [41]. Airway inflammation persists leading to airway damage and respiratory insufficiency, affecting the quality of life, sometimes even causing death. Exposure to environmental factors such as allergens and infections leads to asthma symptoms and morphological changes. The mechanisms of AAHM for asthma have been reported in several studies. Studies have shown that AAHM can significantly reduce asthmatic inflammation by reducing IgE, eosinophils, interleukin 4 (IL-4), and CD4^+^ levels, increasing IL-10 and CD8^+^, and transforming growth factor beta (TGF-*β*) levels [42, 43]. Results of this meta-analysis also show that AAHM has the potential in reducing total IgE and EOS. This suggests that AAHM may improve clinical symptoms by reducing airway hyper-responsiveness and regulating immune function, thus more effectively controlling asthma.

In this review, TCM prescription for acupoint application was a critical factor. The most common points across included studies were Feishu (BL13), Dingchuan (EX-B1), Tiantu (CV22), and Shenshu (BL23). TCM theory suggests that these points tonify the lungs and kidneys, thus preventing asthma. The most commonly used herbs included Xi Xin (Herba Asari), Bai Jie Zi (Semen Brassicae), Gan Sui (Radix Kansui), and Yan Hu Suo (Rhizoma Corydalis). According to TCM theory, these herbs can relieve cough, eliminate phlegm, and have antiasthmatic properties. Additionally, they are pungent and warm in nature, which have the characteristics of diverging and dissipating, causing them to be more easily absorbed through acupoints. These herbs can modulate humoral and cellular immune responses and reduce inflammation [44–46].

In the United States and Canada, the marketing of Xi Xin (Herba Asari) containing drugs is restricted due to aristolochic acid toxicity [47, 48]. In China, Xi Xin is a commonly used remedy for treating colds, cough, and asthma, and there is a strict limit on the amount used in treatments. According to Chinese pharmacopoeia [49], a safe daily dose of oral Xi Xin is 1 to 3 g/d. Nevertheless, overdose can cause serious adverse effects. Another herb commonly used in the studies was Ma Huang (Herba Ephedra), which is restricted in some countries such as Australia but not in other countries such as Japan, Korea, and China, where it is widely used.

It should be noted that despite its popularity in China, Chinese medicine practitioners in other countries do not frequently use acupoints application. This may be due to the commonly observed redness, swelling, and blistering around the acupoint area. In TCM theory, AAHM therapies use herbs with warm or hot properties to irritate the skin. After the continuous stimulation of the herbs, the skin shows redness, heat, and blisters. This is why the most commonly reported adverse event in AAHM studies was skin irritation. However, this is not always considered to be an adverse effect, rather a therapeutic benefit. With this in mind, the safety of AAHM should be cautiously considered. Most of the included studies did not report whether or not adverse events had occurred and studies that did report adverse events judged them to be mild. Therefore, it is unclear the extent of AAHM's adverse event profile and interpretation of adverse events may differ based on region or country of use.

Although this review reveals the positive effects of AAHM for asthma, there remain several limitations that have some impact on the findings. Firstly, the methodology and quality of evidence of the included randomized placebo-controlled trials are still low. Almost half of the included studies did not explicitly report the method of random sequence generation, and the majority of studies did not mention allocation concealment. The results of the GRADE evaluation are mainly very-low- to low-quality evidence. Secondly, the sample sizes of the included studies were relatively small, and power calculations were not performed for the primary outcomes. Finally, most of the included studies did not report adverse events, and there remains insufficient evidence to support the safety of AAHM.

## 5. Conclusion

Evidence from this meta-analysis suggests that AAHM may be an effective treatment for asthma, and it can be performed as an alternative therapy for asthma. AAHM shows great potential in improving the quality of life, improving lung function, and reducing acute exacerbation of asthma. However, the current evidence is not strong enough to routinely recommend AAHM for patients with asthma. The benefits and safety of AAHM still need further evaluation due to study design and reporting weaknesses.

## Figures and Tables

**Table 1:** Characteristics of included studies.

First author, year [ref.]	Country	Age (years, mean ± SD)	Sample size (R/A)	Asthma history Mean ± SD (years)
Yang, 2018 [15]	China	T: 56.67 ± 10.62	T: 30/30	T: 10.17 ± 6.79
		C: 56.80 ± 12.32	C: 30/30	C: 9.87 ± 5.29
Wang, 2017 [16]	China	T: 8.0 ± 2.8	T: 60/60	T: 1.68 ± 0.88
		C: 7.5 ± 2.6	C: 60/60	C: 1.67 ± 0.67
Wang (b), 2017 [17]	China	T:7.6 ± 2.8	T: 50/50	T: 1.58 ± 0.78
		C:7.5 ± 2.6	C: 50/50	C: 1.57 ± 0.72
Ma, 2016 [18]	China	T: 7.6 ± 2.5	T: 54/54	T:2.9 ± 1.4
		C: 8.1 ± 2.7	C: 53/53	C:3.2 ± 1.5
Wang, 2016 [19]	China	T: 48 ± 6.4	T: 44/44	T: 9.5 ± 3.5
		C: 47 ± 6.9	C: 44/44	C: 7.3 ± 3.1
Shi, 2014 [20]	China	T: 4.8 ± 0.3	T: 46/46	T:7.50 ± 3.30
		C: 4.9 ± 0.5	C: 46/46	C:7.30 ± 3.10
Chang, 2014 [21]	China	T: 37.21 ± 12.90	T: 75/75	T:18.28 ± 8.90 M
		C: 38.91 ± 11.85	C: 68/68	C:19.91 ± 9.18 M
Liu, 2018 [22]	China	T: 50 ± 15	T: 30/30	T:8.7 ± 4.7
		C: 49 ± 12	C: 30/30	C:9.1 ± 4.7
Tian, 2020 [23]	China	T: 54.82 ± 11.77	T: 56/56	T: 2–7
		C: 53.6 ± 13.28	C: 55/55	C: 3–7
Shang, 2013 [24]	China	T:35.0 ± 11.0	T: 80/80	T: NR
		C:32.0 ± 12.0	C: 40/40	C: NR
Lu, 2011 [25]	China	T: 46.82 ± 10.11	T: 35/35	T:4.76 ± 1.23
		C: 47.42 ± 11.84	C: 35/35	C:5.42 ± 1.74
Bai, 2010 [26]	China	T: 19–52	T: 42/42	T: 0.25–6
		C: 25–51	C: 42/42	C: 0.16–5
Yao, 2009 [27]	China	T: 45.3 ± 12.0	T: 125/125	T: 12.4 ± 3.4
		C: 45.4 ± 12.7	C: 63/63	C: 11.8 ± 4.7
Yu, 2009 [28]	China	NR	T: 33/33	NR
			C: 17/17	
Yao, 2007 [29]	China	T: 46.2 ± 12.1	T: 44/44	NR
		C: 51.0 ± 11.8	C: 22/22	
Mi, 2005 [30]	China	T: 18–65	T: 181/181	T: 0.16–20
		C: 18–65	C: 181/181	C: 0.08–30

Abbreviations: T: treatment, C: control, R: number of subjects randomized, A: number of subjects analyzed, M: month, NR: not reported, and SD: standard deviation.

**Table 2:** Interventions and outcome assessments.

First author, year (ref.)	Control intervention	Experimental intervention	Treatment duration	Points selection	Outcome
Yang, 2018 [15]	RP + placebo^*∗*^	RP + AAHM Baijiezi (Semen Brassicae), Yanhusuo (Rhizoma Corydalis), Gansui (Radix Kansui), Xixin (Herbal Asari), and Mahuang (Herba Ephedrae); 3 times a year for 2 consecutive years (dog days)	2 years▲	BL15 Xinshu, BL13 Feishu, BL17 Geshu, and BL11 Dazhu	FEV_1_, FEV_1_/FVC, ACT, FAE
Wang, 2017 [16]	RP + placebo^*∗*^	RP + AAHM	1 year▲	BL13 Feishu, BL15 Xinshu, BL17 Geshu, CV17 Danzhong, CV22 Tiantu, and RN8 Shenque	EOS, FEV_1_, IgE
		Baijiezi (Semen Brassicae), Yanhusuo (Rhizoma Corydalis), Gansui (Radix Kansui), Xixin (Herbal Asari), and Shexiang (Moschus)			
		Every 10 days each time and duration for 3 to 4 hours (dog days)			
		Baijiezi (Semen Brassicae), Yanhusuo (Rhizoma Corydalis), Gansui (Radix Kansui), Xixin (Herbal Asari), Shexiang (Moschus), Shudihuang (Radix Rehmanniae Preparata), and Buguzhi (Fructus Psoraleae)			
		Every 9 days each time and duration for 3 to 4 hours (San-Jiu days)			
		Dingxiang (Flos Caryophylli), Sharen (Fructus Amomi), Cangzhu (Rhizoma Atractylodis), Baizhu (White Atractylodes Rhizome), and Hujiao (Fructus Piperis)			
		Duration for 3 to 12 hours (dog days and San-Jiu days)			
Wang, 2017 [17]	RP + placebo^*∗*^	RP + AAHM	3 months★	BL13 Feishu, BL15 Xinshu, BL17 Geshu, CV17 Danzhong, CV22 Tiantu, and RN8 Shenque	FEV_1_
		Baijiezi (Semen Brassicae), Yanhusuo (Rhizoma Corydalis), Gansui (Radix Kansui), Xixin (Herbal Asari), and Shexiang (Moschus)			
		Every 10 days each time and duration for 3 to 4 hours (dog days)			
		Baijiezi (Semen Brassicae), Yanhusuo (Rhizoma Corydalis), Gansui (Radix Kansui), Xixin (Herbal Asari), Shexiang (Moschus), Shudihuang (Radix Rehmanniae Preparata), and Buguzhi (Fructus Psoraleae)			
		Every 9 days each time and duration for 3 to 4 hours (San-Jiu days)			
		Dingxiang (Flos Caryophylli), Sharen (Fructus Amomi), Cangzhu (Rhizoma Atractylodis), Baizhu (White Atractylodes Rhizome), and Hujiao (Fructus Piperis)			
		Duration for 3 to 12 hours (dog days and San-Jiu days)			
Ma, 2016 [18]	Placebo^*∗*^	AAHM Baijiezi (Semen Brassicae), Yanhusuo (Rhizoma Corydalis), Gansui (Radix Kansui), Xixin (Herbal Asari), Rougui (Cortex Cinnamomi), Banxia (Rhizoma Pinelliae), and Shexiang (Moschus); every 3 days each time and duration for 2 hours (dog days)	30 days★	GV14 Dazhui, BL13 Feishu, BL20 Pishu, and BL23 Shenshu	FEV_1_
Wang, 2016 [19]	RP + placebo^*∗*^	RP + AAHM Baijiezi (Semen Brassicae), Yanhusuo (Rhizoma Corydalis), Xixin (Herbal Asari), Gansui (Radix Kansui), and Shexiang (Moschus); every 10 days each time and duration for 2 to 6 hours (dog days)	1 year▲	First dog days:	FEV_1_, IgE
				BL12 Fengmen and BL13 Feishu	
				EX-B1 Dingchuan	
				Middle dog days:	
				BL20 Pishu and BL14 Jueyinshu	
				GV14 Dazhui	
				End dog days:	
				BL23 Shenshu and BL11 Dazhu	
				BL43 Gaohuang	
Shi, 2014 [20]	Placebo^*∗*^	AAHM Baijiezi (Semen Brassicae), Yanhusuo (Rhizoma Corydalis), Gansui (Radix Kansui), Xixin (Herbal Asari), and Fangfeng (Radix Saposhnikoviae); every 10 days each time and duration for 10 minutes (dog days)	1 year▲	BL15 Xinshu, BL13 Feishu, and BL17 Geshu	EOS, FEV_1_, FEV_1_/FVC
Chang, 2014 [21]	Placebo^*∗*^	AAHM	32 days★	Scheme 1: GV14 Dazhui; BL13 Feishu; EX-B1 Dingchuan; CV22 Tiantu; BL12 Fengmen; Scheme 2: BL13 Feishu; BL43 Gaohuang; BL20 Pishu; BL23 Shenshu; CV17 Danzhong.	FAE
		Scheme 1:			
		Baijiezi (Semen Brassicae), Yanhusuo (Rhizoma Corydalis), Gansui (Radix Kansui), Xixin (Herbal Asari), Fangfeng (Radix Saposhnikoviae), Wuweizi (Fructus Schisandrae Chinensis), and Bingpian (Borneclum Syntheticum)			
		Scheme 2:			
		Baijiezi (Semen Brassicae), Yanhusuo (Rhizoma Corydalis), Gansui (Radix Kansui), Xixin (Herbal Asari), Ganjiang (Dried Ginger), Rougui (Cortex Cinnamomi), Yinyanghuo (Herba Epimedii), and Bingpian (Borneclum Syntheticum)			
		(Schemes 1 and 2 were used alternately)			
		Every 4 days each time and duration for 3 to 5 hours (dog days)			
Liu, 2018 [22]	Placebo^*∗*^	AAHM Baijiezi (Semen Brassicae), Yanhusuo (Rhizoma Corydalis), Gansui (Radix Kansui), and Xixin (Herbal Asari); every 10 days each time and duration for 4 hours (dog days)	1 year▲	BL15 Xinshu, BL13 Feishu, and BL17 Geshu	FAE
Tian, 2020 [23]	RP + placebo^*∗*^	RP + AAHM Baijiezi (Semen Brassicae), Yanhusuo (Rhizoma Corydalis), Gansui (Radix Kansui), and Xixin (Herbal Asari); 3 times a year for 2 consecutive years and duration for 2 to 4 hours (dog days)	2 years	BL15 Xinshu, BL13 Feishu, BL17 Geshu, BL20 Pishu, ST40 Fenglong	FAE, FEV_1_
Shang, 2013 [24]	Placebo^*∗*^	AAHM Xixin (Herbal Asari), Baijiezi (Semen Brassicae), Gansui (Radix Kansui), and Mahuang (Herba Ephedrae); twice a week and duration for 1 hour (dog days)	4 weeks★	Along governor vessel and bladder meridian of foot taiyang	IgE
Lu, 2011 [25]	RP + placebo^*∗*^	RP + AAHM Baijiezi (Semen Brassicae), Yanhusuo (Rhizoma Corydalis), Gansui (Radix Kansui), and Xixin (Herbal Asari); every 10 days each time (dog days)	3 years▲	Scheme 1:	FEV_1_, AQLQ, FEV_1_/FVC
				CV22 Tiantu and GV14 Dazhui	
				BL13 Feishu and BL20 Pishu	
				RN8 Shenshu and ST36 Zusanli	
				Scheme 2:	
				CV17 Danzhong and BL42 Pohu ST40 Fenglong and BL49 Yishe	
				EX-B1 Dingchuan and BL52 Zhishi	
				(Schemes 1 and 2 were used alternately)	
Bai, 2010 [26]	Placebo^*∗*^	AAHM Baijiezi (Semen Brassicae), Gansui (Radix Kansui), Fangfeng (Radix Saposhnikoviae), Huangqin (Radix Scutellariae), and Dilong (Pheretima); every 2 or 3 days each time (dog days)	3 years▲	BL23 Shenshu, BL13 Feishu, EX-B1 Dingchuan, BL43 Gaohuang	FAE
Yao, 2009 [27]	Placebo^*∗*^	AAHM Baijiezi (Semen Brassicae), Hujiao (Fructus Piperis), Xixin (Herbal Asari), and Baizhi (Radix Angelicae Dahuricae); every 3 or 4 days each time and duration for 4 to 4 hours (4 weeks)	4 weeks★	Scheme 1:	AQLQ, FEV_1_/FVC
				CV22 Tiantu and GV14 Dazhui;	
				BL13 Feishu and BL20 Pishu	
				Scheme 2:	
				CV22 Tiantu and CV17 Danzhong	
				ST36 Zusanli and RN6 Qihai	
				RN4 Guanyuan	
				(Schemes 1and 2 were used alternately)	
Yu, 2009 [28]	Placebo^*∗*^	AAHM Baijiezi (Semen Brassicae), Hujiao (Fructus Piperis), Xixin (Herbal Asari), Banfenghe (Pterospermum heterophyllum Hance), and Baizhi (Radix Angelicae Dahuricae); twice a week and duration for 4 hours (4 weeks)	4 weeks★	Scheme 1:	FEV_1_, FEV_1_/FVC, AQLQ,
				CV22 Tiantu and GV14 Dazhui	
				BL13 Feishu and BL20 Pishu	
				Scheme 2:	
				CV22 Tiantu and CV17 Danzhong	
				ST36 Zusanli and RN6 Qihai	
				RN4 Guanyuan	
				(Schemes 1 and 2 were used alternately)	
Yao, 2007 [29]	Placebo^*∗*^	AAHM Baijiezi (Semen Brassicae), Hujiao (Fructus Piperis), Xixin (Herbal Asari), Banfenghe (Pterospermum heterophyllum Hance), and Baizhi (Radix Angelicae Dahuricae); twice a week and duration for 4 hours (4 weeks)	4 weeks★	Scheme 1:	EOS, FEV_1_
				CV22 Tiantu and GV14 Dazhui	
				BL13 Feishu and BL20 Pishu	
				Scheme 2:	
				CV22 Tiantu and CV17 Danzhong	
				ST36 Zusanli and RN6 Qihai	
				RN4 Guanyuan	
				(Schemes 1 and 2 were used alternately)	
Mi, 2005 [30]	RP + placebo^*∗*^	RP + AAHM, Baijiezi (Semen Brassicae), Yanhusuo (Rhizoma Corydalis), Gansui (Radix Kansui), and Xixin (Herbal Asari); every 10 days each time and duration for 1 hour (3 months)	3 months★	Scheme 1:	FEV_1_/FVC
				BL13 Feishu and BL21 Weishu	
				CV17 Danzhong and BL52 Zhishi	
				Scheme 2:	
				BL20 Pishu and BL43 Gaohuang	
				BL12 Fengmen and CV22 Tiantu	
				Scheme3:	
				BL23 Shenshu and RN12 Zhongwan	
				EX-B1 Dingchuan and BL23 Xinshu	
				(Schemes 1, 2, and 3 were used alternately)	

Abbreviations: ACT: asthma control test, AAHM: acupoint application of herbal medicine, AQLQ: asthma quality of life questionnaire, FAE: frequency of acute exacerbations, IgE: Immunoglobulin E, RP: routine pharmacotherapy, EOS: eosinophil, FEV_1_: forced expiratory volume in 1 second, and FVC: forced vital capacity. ^*∗*^The application method and point selection are the same as the experimental intervention. ▲Long-term efficacy observation. ★Short-term efficacy observation.

**Table 3:** Summary of GRADE evidence: Comparison of long-term efficacy between AAHM plus RP and placebo plus RP.

Outcomes	Number of participants (number of Studies)	Quality of evidence (GRADE)	Absolute effect	
			Control group (mean)	Comparison between groups（95% CI）
FEV_1_ (%)	449 (5 RCTs)	⊕⊕○○ Low 1,4	70.70%	↑ 11.80 (2.84, 20.76)
FEV_1_/FVC	130 (2 RCTs)	⊕○○○ Very low 1,2,3	67.47%	No statistical significance
FAE	148 (2 RCTs)	⊕○○○ Very Low 2,3,4	3.03 times	No statistical significance
ACT	171 (2 RCTs)	⊕○○○ Very Low 1,2,3	20.71 points	No statistical significance
AQLQ	70 (1 RCT)	⊕⊕⊕○ Moderate 3	109.61 points	↑ 6.53 (2.70, 10.36)
Total IgE	208 (2 RCTs)	⊕○○○ Very Low 1,2,3,4	693.62 IU/ml	No statistical significance
EOS	120 (1 RCT)	⊕⊕○○ Low 3,4	0.61 × 0.09/L	↓ –0.31 (–0.37, –0.25)

Abbreviations: ACT: asthma control test, AQLQ: asthma quality of life questionnaire, FAE: frequency of acute exacerbations, IgE: immunoglobulin E, EOS: eosinophil, FEV_1_: Forced expiratory volume in 1 second, and FVC: forced vital capacity. 1. Considerable statistical heterogeneity, 2. the credible interval contains invalid values and wide interval limits the accuracy of the results, 3. insufficient sample size limits the accuracy of the results, and 4. unclear sequence generation.

**Table 4:** Summary of GRADE evidence: comparison of short-term efficacy between AAHM plus RP and placebo plus RP

Outcomes	Number of participants (number of studies)	Quality of evidence (GRADE)	Absolute effect	
			Control group (mean)	Comparison between groups（95% CI）
FEV_1_ (%)	100 (1 RCT)	⊕⊕○○ Low 3,4	78.61%	↑ 10.57 (8.40, 12.74)
FEV_1_ (L)	88 (1 RCT)	⊕⊕○○ Low 3,4	2.01 L	↑ 0.80 (0.55, 1.05)
FEV_1_/FVC	362 (1 RCT)	⊕⊕○○ Low 3,4	72.11%	↑ 7.77 (6.07, 9.47)

Abbreviations: ACT: asthma control test, AQLQ: asthma quality of life questionnaire, FAE: frequency of acute exacerbations, IgE: immunoglobulin E, EOS: eosinophil, FEV_1_: forced expiratory volume in 1 second, and FVC: forced vital capacity. 1. Considerable statistical heterogeneity, 2. the credible interval contains invalid values and wide interval limits the accuracy of the results, 3. insufficient sample size limits the accuracy of the results, and 4. unclear sequence generation.

**Table 5:** Summary of GRADE evidence: comparison of long-term efficacy between AAHM and placebo

Outcomes	Number of participants (number of Studies)	Quality of evidence (GRADE)	Absolute effect	
			Control group (mean)	Comparison between groups（95% CI）
FEV_1_ (L)	66 (1 RCT)	⊕⊕○○ Low 3,4	1.64 L	↑ 0.35 (0.03, 0.67)
FEV_1_/FVC	92 (1 RCT)	⊕⊕○○ Low 2,3	79.86%	↑ 11.75 (4.04, 19.46)
FAE	144 (2 RCTs)	⊕○○○ Very Low 1,3,4	3.57 times	↓–1.52 (–2.17, –0.87)
EOS	92 (1 RCT)	⊕⊕○○ Low 2,3	0.76 × 0.09/L	No statistical significance

Abbreviations: AQLQ: Asthma quality of life questionnaire; FAE: Frequency of acute exacerbations; IgE: Immunoglobulin E; EOS: Eosinophil; FEV_1_: Forced expiratory volume in 1s; FVC: forced vital capacity. 1. Considerable statistical heterogeneity; 2. The credible interval contains invalid values and wide interval limits the accuracy of the results; 3. Insufficient sample size limits the accuracy of the results; 4. Unclear sequence generation.

**Table 6:** Summary of GRADE evidence: comparison of short-term efficacy between AAHM and placebo

Outcomes	Number of participants (number of studies)	Quality of evidence (GRADE)	Absolute effect	
			Control group (mean)	Comparison between groups（95% CI）
				
FEV_1_ (%)	238 (2 RCTs)	⊕○○○ Very low 1,2,3	78.55%	No statistical significance
FEV_1_ (L)	107 (1 RCT)	⊕⊕○○ Low 3,4	1.46 L	↑ 0.66 (0.59, 0.73)
FEV_1_/FVC	238 (2 RCTs)	⊕○○○ Very low 1,2,3	71.07%	No statistical significance
FAE	143 (1 RCT)	⊕⊕⊕○ Moderate 3	2.74 times	↓ –1.29 (–1.66, –0.93)
AQLQ	238 (2 RCT)	⊕⊕⊕○ Moderate 3	107.58 points	↑ 6.57 (3.76, 9.38)
Total IgE	120 (1 RCT)	⊕⊕○○ Low 3,4	208.50 IU/ml	↓ –104.40 (–113.14, –95.66)
EOS	66 (1 RCT)	⊕⊕○○ Low 2,3	0.31 × 109/L	No statistical significance

Abbreviations: AQLQ: asthma quality of life questionnaire, FAE: frequency of acute exacerbations, IgE: immunoglobulin E, EOS: eosinophil, FEV_1_: forced expiratory volume in 1 second, and FVC: forced vital capacity. 1. Considerable statistical heterogeneity, 2. the credible interval contains invalid values and wide interval limits the accuracy of the results, 3. insufficient sample size limits the accuracy of the results, and 4. unclear sequence generation.

## Data Availability

All data of this review are available from the public, open-access electronic databases.

## Abbreviations

95% CI: 95% Confidence interval
AAHM: Acupoint application of herbal medicine
ACT: Asthma control test
AMED: Allied and Complementary Medicine Database
AQLQ: Asthma quality of life questionnaire
CBM: Chinese Biomedical Database
CINAHL: Cumulative Index of Nursing and Allied Health Literature
CENTRAL: Cochrane Central Register of Controlled Trials
CNKI: China National Knowledge Infrastructure
CQVIP: ChongQing VIP
Embase: Excerpta Medica Database
FEV_1_: Forced expiratory volume in 1 second
FVC: Forced vital capacity
GINA: Global Initiative for Asthma
IgE: Immunoglobulin E
MCID: Minimal clinically important difference
MD: Mean difference
PEF: PEAK expiratory flow
RCTs: Randomized controlled trials
RevMan: Review Manager
RP: Routine pharmacotherapy
TCM: Traditional Chinese medicine
WFMO: Wanfang Medicine Online.

## Supplementary Materials

Supplementary Materials Figure S1. Risk of bias graph. Figure S2. Forest plot of treatment group versus control group: FEV_1_ (L). Figure S3. Forest plot of treatment group versus control group: FEV_1_/FVC. Figure S4. Forest plot of treatment group versus control group: total IgE. Figure S5. Forest plot of treatment group versus control group: EOS (×10^9^/L).
