# Supplementary material for: The Role of Acupoint Application of Herbal Medicine for Asthma: Meta-Analysis of Randomized Double-Blind Placebo-Controlled Trials
Source: Evid Based Complement Alternat Med. 2022 Jan 31;2022:5589433. doi: 10.1155/2022/5589433 (PMC9477641; doi:10.1155/2022/5589433)
Supplement: Supplementary Materials — Figure S1. Risk of bias graph. Figure S2. Forest plot of treatment group versus control group: FEV1 (L). Figure S3. Forest plot of treatment group versus control group: FEV1/FVC. Figure S4. Forest plot of treatment group versus control group: total IgE. Figure S5. Forest plot of treatment group versus control group: EOS (×109/L). [file 5589433.f1.docx]

**SUPPLEMENTARY INFORMATION**

**TITLE:** **The Role of Acupoint Application of Herbal Medicine for Asthma: Meta-analysis of Randomized Double-blind Placebo-controlled Trials**

(*List available Supplementary Information items in the following order*)

*
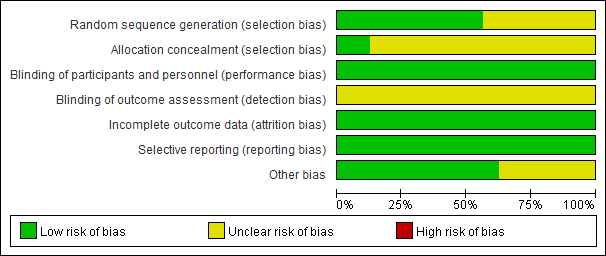
*

**Figure S1.** *Risk of bias graph*


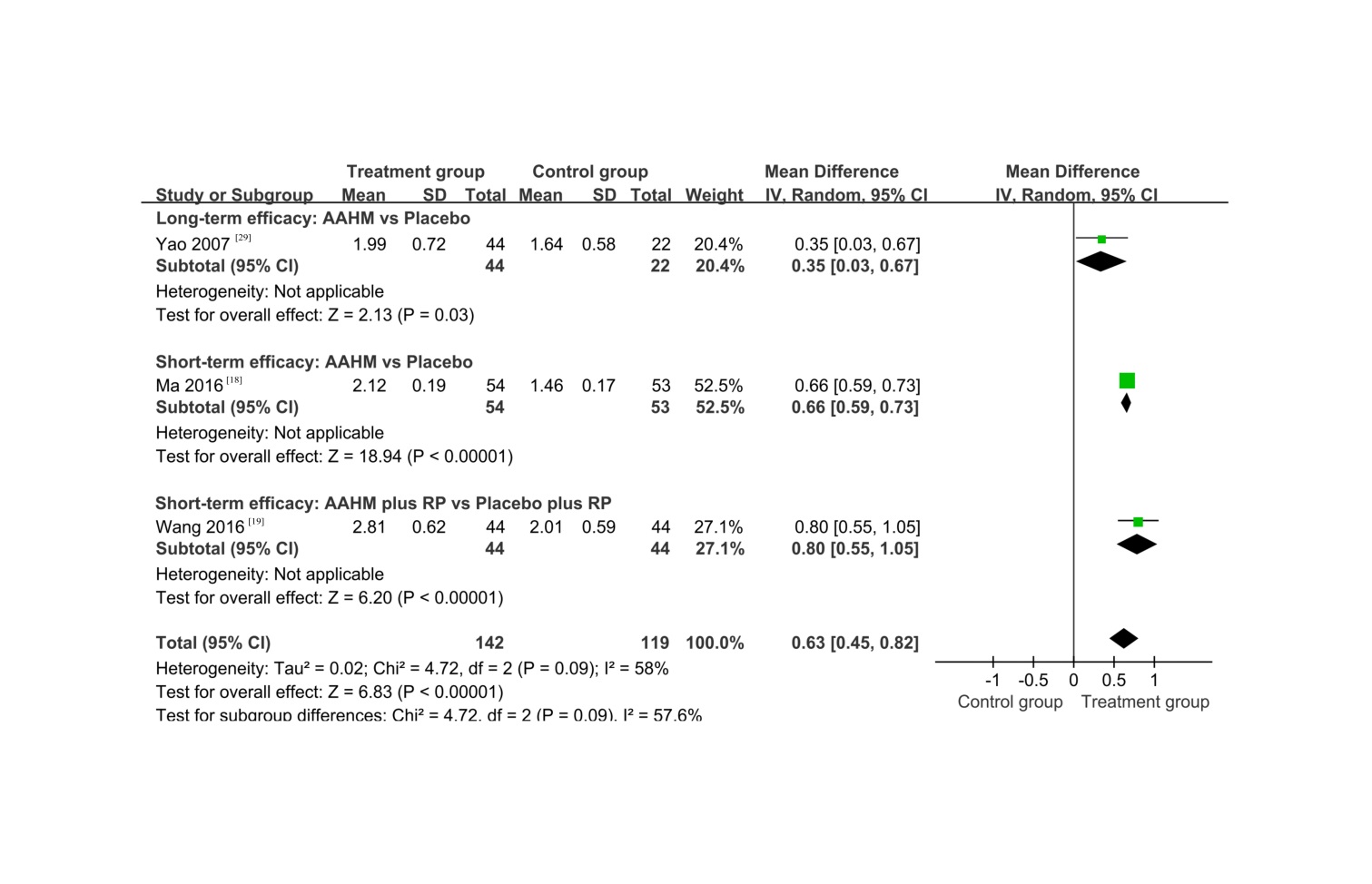


**Figure S2.** *Forrest plot of treatment group versus control group: FEV_1_ (L)*

**
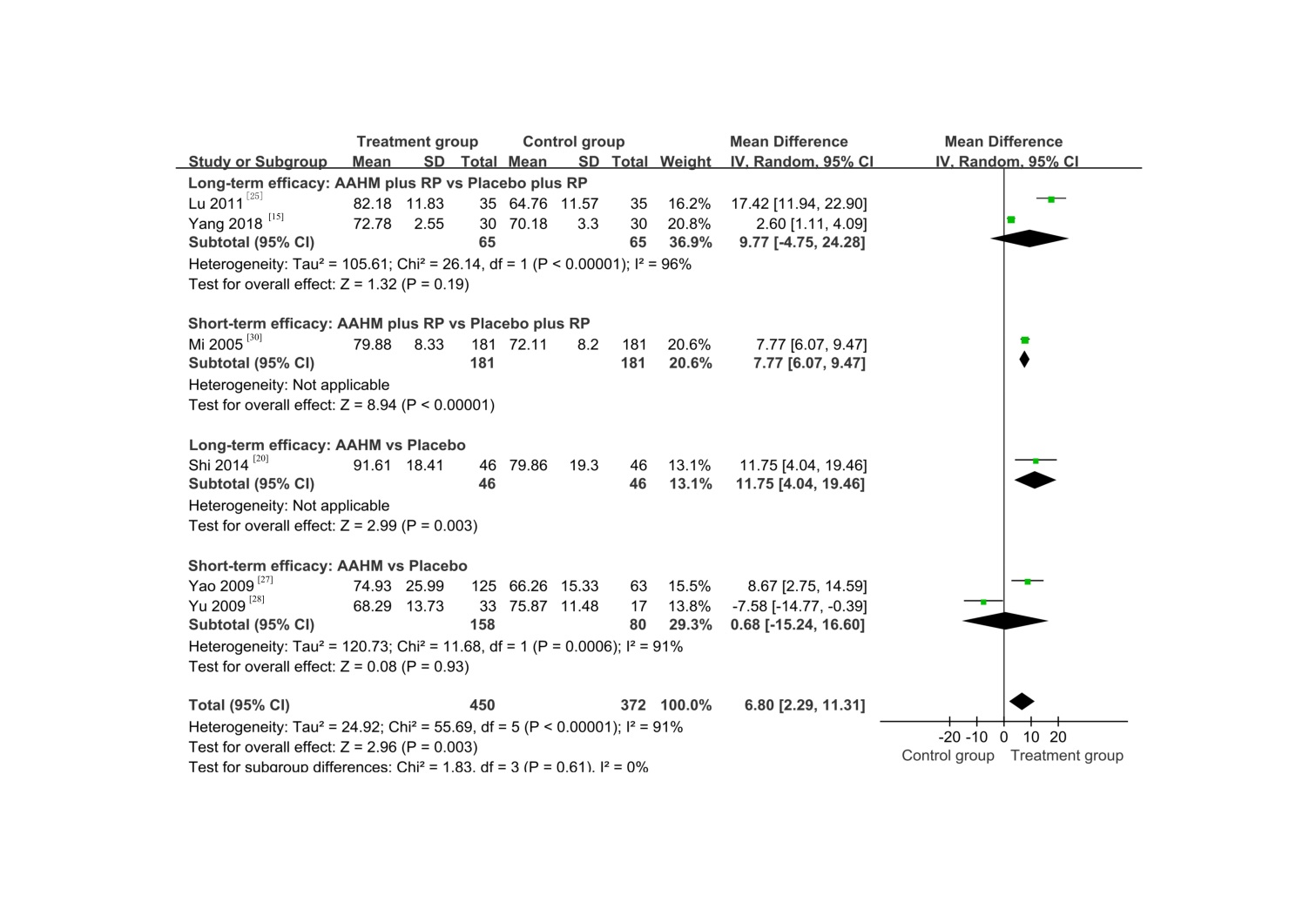
**

**Figure S3.** *Forrest plot of treatment group versus control group: FEV_1_ / FVC*

**
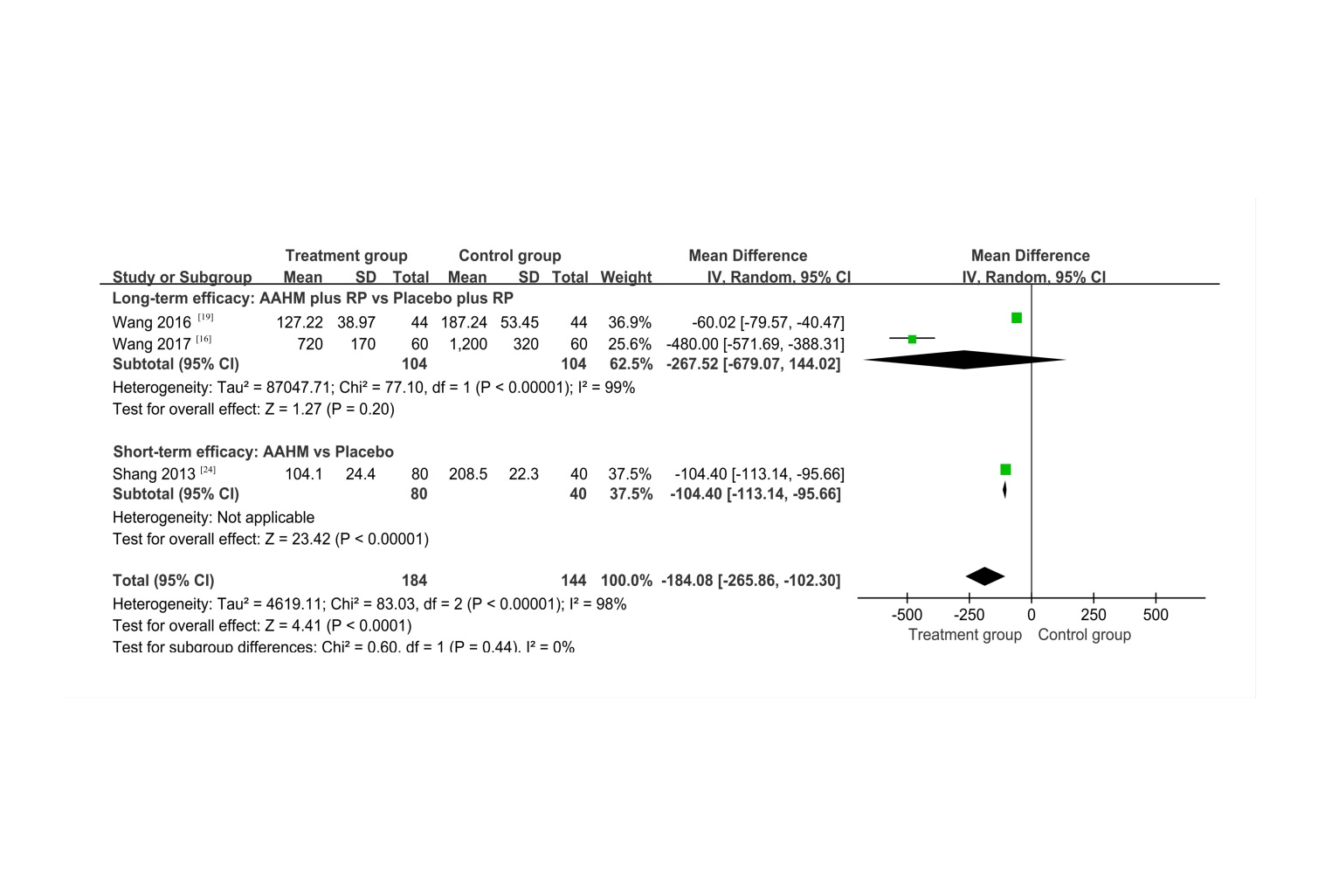
**

**Figure S4.** *Forrest plot of treatment group versus control group: Total IgE*

**
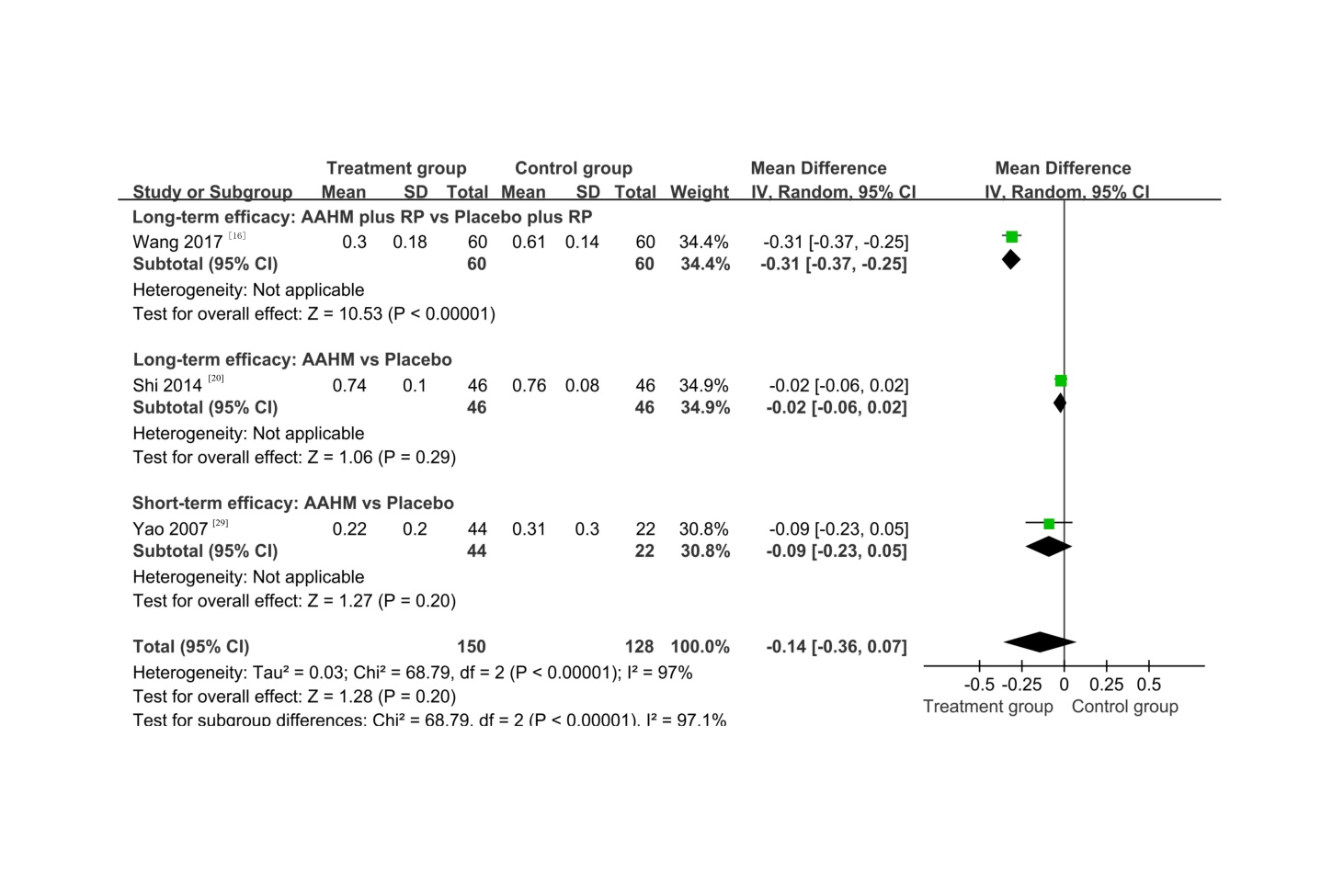
**

**Figure S5.**  *Forrest plot of treatment group versus control group: EOS (×10^9^/L)*
